# Endothelial dysfunction and angiogenesis: what is missing from COVID-19 and cannabidiol story?

**DOI:** 10.1186/s42238-022-00129-w

**Published:** 2022-04-12

**Authors:** Nazanin Ashtar Nakhaei, Andisheh Najarian, Mohammad Hosein Farzaei, Amir Hossein Norooznezhad

**Affiliations:** 1 School of Medicine, Tehran University of Medical Sciences, Tehran, Iran; 2 Medical Biology Research Center, Health Technology Institute, Kermanshah University of Medical Sciences, Kermanshah, Iran; 3 Pharmaceutical Sciences Research Center, Health Institute, Kermanshah University of Medical Sciences, Kermanshah, Iran

**Keywords:** Coronavirus disease 2019, Cannabidiol, Angiogenesis, Endothelial cells, Inflammation

## Abstract

**Background:**

Among pathways involved in the pathogenesis of coronavirus disease 2019 (COVID-19), impaired endothelial cell (EC) function and angiogenesis have been discussed less frequently than others such as cytokine storm. These two do play parts in the development of various clinical manifestations of COVID-19 including acute respiratory distress syndrome (ARDS) and the hyper-coagulation state.

**Methods:**

This narrative review attempts to gather recent data on the possible potential of cannabidiol in the treatment of COVID-19 with an eye on angiogenesis and endothelial dysfunction. Keywords including cannabidiol AND angiogenesis OR endothelial cell as well as coronavirus disease 2019 OR COVID-19 AND angiogenesis OR endothelial dysfunction were searched among the databases of PubMed and Scopus.

**Results:**

Cannabidiol (CBD), as a therapeutic phytocannabinoid, has been approved by the Food and Drug Administration (FDA) for two types of seizures. Due to the potent anti-inflammatory properties of CBD, this compound has been suggested as a candidate treatment for COVID-19 in the literature. Although its potential effect on ECs dysfunction and pathologic angiogenesis in COVID-19 has been overlooked, other than cytokines like interleukin 1β (IL-β), IL-6, IL-8, and tumour necrosis factor α (TNFα) that are common in inflammation and angiogenesis, CBD could affect other important factors related to ECs function and angiogenesis. Data shows that CBD could decrease pathologic angiogenesis via decreasing ECs proliferation, migration, and tube formation. These activities are achieved through the suppression of vascular endothelial growth factor (VEGF), platelet-derived growth factor (PDGF), urokinase plasminogen activator (uPA), matrix metalloproteinase 2 (MMP-2), MMP-9, intracellular adhesion molecule 1 (ICAM-1), and vascular cell adhesion molecule-1 (VCAM-1). Moreover, in an animal model, ARDS and sepsis responded well to CBD treatment.

**Conclusion:**

Altogether and considering the current use of CBD in the clinic, the conduction of further studies on CBD administration for patients with COVID-19 seems to be useful.

## Background

It has been almost two years since the official announcement of Coronavirus Disease 2019 (COVID-19) as a global emergency by the World Health Organization (WHO) (Norooznezhad et al. 2020b). This disease which mainly involves the lungs with the main presentation of pneumonia could also have different other clinical manifestations such as acute respiratory distress syndrome, septic shock, sepsis (Hantoushzadeh and Norooznezhad 2020), and thromboembolic events (Malas et al. 2020). From a pathophysiological point of view, cytokine storm or the excessive release of proinflammatory cytokines is very crucial to the pathogenesis of many morbidities and even mortality in patients diagnosed with COVID-19 (Mahmudpour et al. 2020). However, other new pathways also suggested to be involved in the pathophysiology of COVID-19 among which are endothelial cell (EC) dysfunction and pathological angiogenesis (Hariri and Hardin 2020; Ackermann et al. 2020; Norooznezhad and Mansouri 2021). These have also earned notable as potential targets for new treatment options.

Cannabinoids are mostly known as the active compounds of *Cannabis Sativa* with an affinity to their two specific G protein-coupled receptors: CB1 and CB2. These chemical agents could be categorized into two main groups based on their sources of either endocannabinoids or exocannabinoids, with the latter being divided into plant-derived (phytocannabinoids) and synthetic cannabinoids (Norooznezhad and Norooznezhad 2017; Shahbazi et al. 2020). Cannabidiol (CBD) is a non-psychoactive phytocannabinoid that acts as an antagonist for CB1 and CB2 receptors at low nanomolar concentrations but as an agonist/inverse agonist at micromolar concentrations (Stanley et al. 2013). Recent studies have suggested CBD as a possible treatment for COVID-19 while merely focusing on the inflammation aspect (Costiniuk and Jenabian 2020). However, COVID-19 is much more complicated than just an inflammatory outburst, especially in severe cases. Fortunately, with all the recent evidence published on the disease, there is more data now on the pathophysiology of COVID-19. In this narrative review, the possible potential of CBD for the treatment of patients with severe COVID-19 would be evaluated from both angiogenesis and endothelial dysfunction perspectives.

## Methods

This narrative review gathers recent data on the possible potential of cannabidiol in the treatment of COVID-19 with an eye on angiogenesis and endothelial dysfunction. Keywords included cannabidiol AND angiogenesis OR endothelial cell as well as coronavirus disease 2019 OR COVID-19 AND angiogenesis OR endothelial dysfunction and searched databases consisted of PubMed and Scopus with no time limitation (search time: September 2021). The authors separated and extracted the results through two steps: first, reading the title/abstract of articles, and in case of matching with the primary concept of review, the full text was read and extracted.

### Concept

Cannabinoids are well-known for their anti-angiogenic and anti-inflammatory potentials (Norooznezhad and Norooznezhad 2017; 2016; Norooznezhad et al. 2016; Baban et al. 2021). So far, some studies have suggested these compounds as a possible treatment for COVID-19 among which none have discussed their potential to inhibit endothelial cell dysfunction or pathologic angiogenesis (Rossi et al. 2020; Costiniuk and Jenabian 2020; Mohammed et al. 2020; Malinowska et al. 2021). Regarding the role of angiogenesis and endothelial dysfunction in the pathogenesis of COVID-19 and the already mentioned potent anti-inflammatory properties of CBD, it seems that this compound might be effective in the treatment of severe COVID-19 by affecting multiple pathways.

### Angiogenesis and endothelial dysfunction in COVID-19

Recently, upon our review research on the role of angiogenesis and endothelial dysfunction in COVID-19 (Norooznezhad and Mansouri 2021), we found that among many EC surface receptors, angiotensin-converting enzyme 2 (ACE2) receptor associates most with COVID-19 infection. As it has been shown, severe acute respiratory syndrome coronavirus 2 (SARS-CoV-2) infects the endothelial cells using this receptor (Varga et al. 2020). Also, autopsies have revealed higher numbers of ACE2 positive endothelial cells in the lungs of COVID-19 patients compared to healthy cases (Ackermann et al. 2020).

Ackerman et al., have shown that pathologic angiogenesis, as well as micro thromboembolism, occurs in non-survivor COVID-19 patients. Moreover, through their investigation, endothelialitis and endothelial cell dysfunction were observed as well in the patients diagnosed with SARS-CoV-2 infection. As they have stated, the expression of pro-angiogenic and pro-inflammatory factors involved in angiogenesis is significantly increased in patients with COVID-19 compared to the uninfected control tissues. Among these factors, hypoxia-inducible factor 1α (HIF-1α), vascular endothelial growth factor (VEGF), matrix metalloproteinase 2 (MMP-2), interleukin 6 (IL-6), insulin-like growth factor 1 (IGF-1), and FLT-1 or VEGF receptor 1 were more notable (Ackermann et al. 2020).

In another study, it was shown that plasma levels of VEGF-A, platelet-derived growth factor AA (PDGF-AA), and PDGF-AB/BB in non-intensive care unit (ICU) patients were significantly higher than controls. Also, in expired ICU admitted patients, the levels of plasminogen activator inhibitor-1 (PAI-1), follistatin, and angiopoietin 2 (Ang2) were found to be significantly higher than those of survivors. Moreover, Ang2, endoglin, fibroblast growth factor (FGF), FLT-3L, and PAI-1 were significantly higher in ICU admitted patients compared to other COVID-19 individuals (Pine et al. 2020). According to another investigation, COVID-19 patients with increased D-dimer and C reactive protein (CRP) levels as well as creatinine, lymphopenia, and decreased SpO_2_ levels experienced significant levels of soluble E-selectin and Ang2 (Smadja et al. 2020).

### Cannabidiol, angiogenesis, and endothelial cells

As mentioned, cannabinoids have been studied for their strong anti-inflammatory potentials and ability to inhibit/decrease various important pro-inflammatory cytokines such as IL-1β, IL-6, and TNF-α. Other than the anti-inflammatory potential, cannabinoids also have shown notable anti-angiogenic potential (Norooznezhad and Norooznezhad 2016, 2017; Norooznezhad et al. 2016; Mansouri and Norooznezhad 2019). Cannabinoids exert their biological activity through two main G protein-coupled receptors of CB1 and CB2 (Shahbazi et al. 2020). These receptors are expressed in a wide range of tissues and cells. Among the cannabinoids, some have an affinity to one or both of these receptors rendering them an agonist or antagonist potential. The CB1 receptor is expressed on smooth muscle cells of arterioles (Howlett and Abood 2017), endothelial cells (Liu et al. 2000), and certain other cells and tissues (Howlett and Abood 2017). CB2 receptor, with no psychoactive properties, has also been found on the vascular endothelial cells (Blázquez et al. 2003) as well as many other organs and tissues (Howlett and Abood 2017).

As Solinas et al., have demonstrated, CBD could inhibit endothelial cell tube formation and morphogenesis by decreasing cellular proliferation without inducing apoptosis or exerting any toxic effect. Also, one of the other inhibited pathways of angiogenesis by CBD is endothelial cells migration. In this regard, CBD has been illustrated to decrease the expression of *uPA*, *MMP-9*, *PAI-1*, *PDGF-AA*, and *IL-8* (up to 50%) in human endothelial cells (Solinas et al. 2012). Furthermore, investigations have cleared that CBD could decrease *vascular cell adhesion molecule-1* (*VCAM-1*) and *intracellular adhesion molecule-1* (*ICAM-1*) expression, monocytes adhesion potential to endothelial cells, the endothelial barrier integrity, mitochondrial superoxide generation, and activation of NF-κB in high glucose-treated endothelial cells (Rajesh et al. 2007). Also, another study on a CBD hydroxyquinone (HU-331) showed the higher potential of this specific cannabinoid on the inhibition of angiogenesis (considering total vessel number, length, and area) induced by VEGF and FGF. HU-331 is also known to be able to decrease the von Willebrand factor (VWF) in human endothelial cells (Kogan et al. 2006). According to the studies, CBD has been identified to inhibit heparin cocktail-induced angiogenesis through suppression of VEGF and TNF-α. This agent also inhibits endothelial cell proliferation (inducing cytostasis but not apoptosis), migration, and invasion by suppressing different angiogenic factors such as PDGF, MMP-2, MMP-9, and IL-8 (Solinas et al. 2012). Also, in a viral in vivo model, CBD was demonstrated to decrease inflammation by affecting endothelial cells via VCAM-1, an endothelial-expressed protein for leukocytes transmigration (Mecha et al. 2013). It is also believed that CBD could increase the human mesenteric artery vasorelaxation. Interestingly, CBD can decrease NF-κB and phosphorylated c-Jun N-terminal kinases (JNK); thus, preventing the phosphorylation of eNOS (Stanley et al. 2015). In a diabetic model of cardiac dysfunction, CBD treatment has resulted in cardiac function improvement through inhibition of NF-κB leading to decreased *VCAM-1*, *ICAM-1*, and *TNF-α* expressions as well as decreasing other agents involved in oxidative stress (Rajesh et al. 2010). Altogether, it seems that other than affecting endothelial cells in the cardiovascular system, CBD could positively improve cardiac function in patients with COVID-19. Similar to its cardioprotective effects, CBD could show protective properties in other organs such as the kidney, liver, and nervous systems as well (Malinowska et al. 2021).

**Fig. 1:**
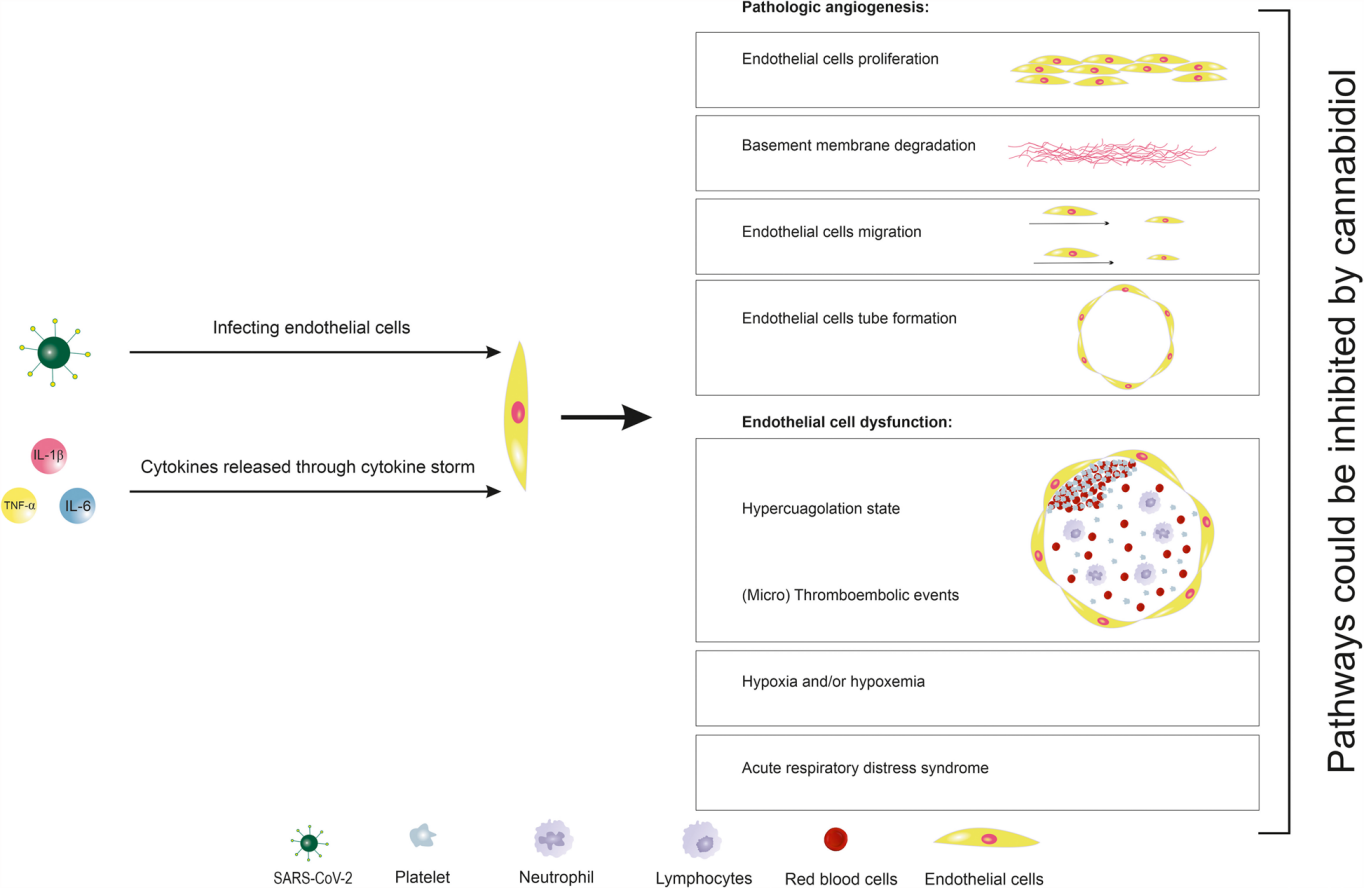
Short review on the potential of cannabidiol on pathologic angiogenesis and endothelialitis. Severe acute respiratory syndrome coronavirus 2 (SARS-CoV-2) infects the endothelial cells and led to pathologic angiogenesis and endothelial dysfunction. These features could be inhibited by using cannabidiol treatment. IL, interleukin; TNF-α, tumor necrosis factor α

Endothelial cell dysfunction and some of the pro-angiogenic factors are directly associated with ARDS (Ackermann et al. 2020; Norooznezhad and Mansouri 2021). A recent *in vivo* study on viral infection-induced ARDS evaluated the outcomes of CBD treatment. It has been shown that CBD not only increases O_2_ saturation to the normal value but also reduces IL-6, TNF-α, and INF-γ levels. Another interesting point in this study was the reduced neutrophil and increased lymphocytes count following CBD treatment (Khodadadi et al. 2020). As has been demonstrated, increased neutrophils to lymphocytes ratio (NLR) as well as lymphopenia alone are among the poor prognostic factors in patients with COVID-19 (Norooznezhad et al. 2020a; Terpos et al. 2020). Also, it has been shown that the administration of CBD has decreased pro-inflammatory cytokines (e.g. IL-6 and TNF-α) in an animal model of ARDS (Khodadadi et al. 2020). All in all, it seems that CBD could be considered as an effective treatment option functioning through the most important pathways involved in pathologic angiogenesis and endothelial cell dysfunction. Figure 1 shows some of the possible potentials of CBD on angiogenesis and endothelial dysfunction in SARS-CoV-2 infection.

### Clinical use of cannabidiol

As already mentioned, CBD is a non-psychoactive phytocannabinoid that has been used in many trials for a wide variety of diseases. In Jun 2018, the Food and Drug Administration (FDA) of the United States approved an oral solution of CBD under the brand name of Epidiolex® for two rare forms of epilepsy, Lennox-Gastaut and Dravet syndromes or tuberous sclerosis complex (TSC) in young children 1 year of age and older. Upon this decision, Epidiolex® became the first FDA-approved cannabinoid in history. (Brunetti et al. 2020). Recently, a pilot randomized, parallel-arm, double-blind investigation has been performed on the anti-inflammatory activity of CBD in healthy adults. After the administration of CBD, their peripheral blood mononuclear cells (PBMCs) were harvested and cultured with lipopolysaccharide (LPS). A significant decrease was observed in TNF-α levels from their PBMCs upon previous exposure to CBD compared to the cells collected before CBD treatment (Hobbs et al. 2020). However, similar to any other medication, CBD has certain side effects which are considered not serious. Among the described side effects of CBD, somnolence, fatigue, hepatic abnormalities, vomiting, and diarrhoea are the most prevalent (Huestis et al. 2019). Also, drug-drug interaction with COVID-19 medications should also be kept in mind while using CBD (Malinowska et al. 2021). It is noteworthy to mention that a combination of CBD and THC (Sativex; THC: CBD in 1:1 ratio) has been approved for the treatment of MS-associated spasticity in 25 countries except for the US (Khalsa et al. 2021).

## Conclusion

Other than cytokine storm, pathologic angiogenesis and endothelial cell dysfunction are critical to the pathogenesis and outcomes of COVID-19. In this review, the most important molecular pathways of angiogenesis and endothelial cell dysfunction were suggested to be targeted by CBD for the treatment of COVID-19. Herein, we emphasize that a significant amount of research from clinical studies to trials is needed before CBD could be further developed into a medicine and prescribed for treating any of the promoted clinical conditions including the complications of COVID-19.

## Data Availability

Not applicable.

## Abbreviations

CBD: Cannabidiol
COVID-19: Coronavirus disease 2019
EC: Endothelial cell
ACE2: Angiotensin-converting enzyme 2
PAI-1: Plasminogen activator inhibitor-1
Ang2: Angiopoietin 2
HIF-1α: Hypoxia-inducible factor 1α
VEGF: Vascular endothelial growth factor
MMP-2: Matrix metalloproteinase 2
IL-6: Interleukin 6
IGF-1: Insulin-like growth factor 1
